# Cancer‐related outcomes in kidney allograft recipients in England versus New York State: a comparative population‐cohort analysis between 2003 and 2013

**DOI:** 10.1002/cam4.1015

**Published:** 2017-01-30

**Authors:** Francesca Jackson‐Spence, Holly Gillott, Sanna Tahir, Jay Nath, Jemma Mytton, Felicity Evison, Adnan Sharif

**Affiliations:** ^1^ University of BirminghamBirminghamUnited Kingdom; ^2^ Department of Nephrology and TransplantationQueen Elizabeth HospitalEdgbastonBirminghamUnited Kingdom; ^3^ Department of Health InformaticsQueen Elizabeth HospitalEdgbastonBirminghamUnited Kingdom

**Keywords:** Cancer, epidemiology, geography, kidney transplant

## Abstract

It is unclear whether cancer‐related epidemiology after kidney transplantation is translatable between countries. In this population‐cohort study, we compared cancer incidence and all‐cause mortality after extracting data for every kidney‐alone transplant procedure performed in England and New York State (NYS) between 2003 and 2013. Data were analyzed for 18,493 and 11,602 adult recipients from England and NYS respectively, with median follow up 6.3 years and 5.5 years respectively (up to December 2014). English patients were more likely to have previous cancer at time of transplantation compared to NYS patients (5.6% vs. 3.5%, *P *< 0.001). Kidney allograft recipients in England versus NYS had increased cancer incidence (12.3% vs. 5.9%, *P *< 0.001) but lower all‐cause mortality during the immediate postoperative stay (0.7% vs. 1.0%, *P *= 0.011), after 30‐days (0.9% vs. 1.8%, *P *< 0.001) and after 1‐year post‐transplantation (3.0% vs. 5.1%, *P *< 0.001). However, mortality rates among patients developing post‐transplant cancer were equivalent between the two countries. During the first year of follow up, if patients had an admission with a cancer diagnosis, they were more likely to die in both England (Odds Ratio 4.28 [95% CI: 3.09–5.93], *P *< 0.001) and NYS (Odds Ratio 2.88 [95% CI: 1.70–4.89], *P *< 0.001). Kidney allograft recipients in NYS demonstrated higher hazard ratios for developing kidney transplant rejection/failure compared to England on Cox regression analysis. Our analysis demonstrates significant differences in cancer‐related epidemiology between kidney allograft recipients in England versus NYS, suggesting caution in translating post‐transplant cancer epidemiology between countries.

## Introduction

The need for lifelong immunosuppression to prevent rejection of the kidney allograft is associated with significant complications for patients after kidney transplantation 1. Perhaps of greatest significance in the contemporary era of immunosuppression is the development of cancer 2, which is increasingly recognized as one of the primary causes of mortality post kidney transplantation 3, 4, 5 and one of the leading causes of concern from a patients’ perspective 6. Therefore, efforts to increase our understanding of cancer‐related epidemiology after kidney transplantation should be actively encouraged to aid transplant‐specific clinical management and patient counseling.

However, our understanding of cancer incidence post kidney transplantation is limited to a small selection of population‐cohort analyses specific to North America, Australia/New Zealand or the United Kingdom 7, 8, 9, 10, 11. Transplant clinicians frequently rely on data published from population‐cohort studies conducted in one country and translate directly to another. However, cancer‐related epidemiology may not be translatable between countries with developed kidney transplantation programs such as England and the United States for a number of reasons. From a transplant perspective kidney allograft recipient phenotypes differ between England and the United States with regard to race, use of T‐cell depletion as induction therapy and utilization of extended‐criteria kidneys, all of which impact upon risk for cancer 12, 13, 14. From a more generic perspective, England demonstrates significantly less self‐reported illnesses and biological markers of disease compared to the United States despite less than half the expenditure per person 15. However, from a cancer perspective, England appears to have a lower incidence but higher mortality from cancer compared to the United States 16. Therefore, we can speculate that cancer‐reported incidence and outcomes after kidney transplantation may not be translatable between countries in the context of significant imbalance in healthcare systems, patient demographics and nature of disease.

To investigate this further, and to determine whether country‐specific cancer epidemiology post‐transplantation is translatable between countries, we undertook a comparative population‐cohort study between England and New York State (NYS) over a contemporaneous era. The aim was to ascertain if cancer‐related epidemiology for kidney allograft recipients differ between the two countries to better inform clinical practice and guide targeted patient counseling.

## Subjects and Methods

### Study population

We obtained data from every kidney‐alone transplant procedure performed in England and New York State between 2003 and 2013, collecting patient demographics that included age, gender, donor type (living or deceased), transplant year, medical comorbidities (based upon ICD‐10 codes) and ethnicity. English data were obtained from Hospital Episode Statistics 17, an administrative data warehouse containing admissions to all National Health Service hospitals in England. It contains detailed records relating to individual patient treatments; with data extraction facilitated utilizing codes on procedural classifications (Office of Population Censuses and Surveys Classification of Interventions and Procedures, 4th revision [OPCS‐4]) 18 and medical classifications (World Health Organization International Classification of Disease, 10th revision [ICD‐10]) 19. The comparative analysis with the United States was performed with contemporaneous New York State data and extracted from the Statewide Planning and Research Cooperative System (SPARCS), a comprehensive all payer data reporting system collecting patient‐level data across New York State 20. The database collects information including patient demographics, diagnoses, procedures, and charges for every inpatient hospital admission, ambulatory surgical procedure, and emergency department admission. Individuals are assigned a unique, encrypted identification code, allowing for longitudinal analyses. Estimated reporting completeness obtained from SPARCS inpatient annual reports during the study period (2000‐11) ranged from 95% to 100%, with an average of more than 98%.

This study included all kidney transplant procedures (OPCS‐4 codes; M01[0‐5,8,9]) performed in England and New York State between the years of 2003 to 2013. Cancer was defined as any post‐transplant admission with ICD‐10 codes C00‐C96, while pretransplant cancer was diagnosed with appropriate ICD‐10 codes listed at time of kidney transplantation. With regard to outcome analysis, both HES and SPARCS data sets have the limitation of only capturing deaths occurring in a hospital setting. To obtain the complete mortality list, the study cohort was cross‐referenced with mortality data from the Office for National Statistics and New York City/New York State vital statistics, respectively, which collects information on all registered deaths in the United Kingdom and New York State respectively. Combining sources via this data linkage process creates a comprehensive dataset with regard to mortality, which was the endpoint of interest in this analysis. This study did not require institutional review board approval due to the pseudoanonymized nature of the data retrieved—data were linked by NHS Informatics utilizing a special HES ID code and avoided patient identifiable codes. With regard to outcome analysis, both HES and SPARCS datasets have the limitation of only capturing deaths occurring in a hospital setting. To obtain the complete mortality list, the study cohort was cross‐referenced with mortality data from the Office for National Statistics and New York State/New York City Vital Statistics respectively, which collects information on all registered deaths in the United Kingdom and New York State, respectively. Combining sources via this data linkage process creates a comprehensive dataset with regard to mortality, which was the endpoint of interest in this analysis. In addition, we extracted data for transplant rejection/failure (ICD‐10; T861 or ICD‐9; 99681). This study did not require institutional review board approval due to the pseudoanonymized nature of the data retrieved—data were linked by NHS Informatics utilizing a special HES ID code and avoided patient identifiable codes.

### Data inclusion

We extracted data on all kidney allograft recipients between the dates of 1^st^ January 2003 and 31st December 2013, who underwent their kidney transplant procedure in a transplant center in either England or New York State. We excluded the following patients from analysis; missing demographic data, combined solid‐organ transplant, and pediatric cases (aged under 18 years).

### Statistical analysis

The primary outcome measures were cancer‐related incidence after kidney transplantation and the risk for mortality (including in‐hospital death, 30‐day and 1‐year mortality) and kidney transplant failure.

Differences between groups were compared using chi‐square tests for categorical variables and Mann–Whitney tests for all continuous variables. Survival analyses were performed, where time to event data was available, using Cox's proportional hazards model and the generation of Kaplan–Meier plots. Otherwise multivariate logistic regression models were used. Variables included in the model were ethnicity, age, gender, donor type (living vs. deceased), year of transplant and selected medical comorbidities (history of myocardial infarction, peripheral vascular disease, cerebrovascular disease, congestive cardiac failure pulmonary disease, liver disease, peptic ulcer, previous cancer and diabetes). Recipients were identified as having allograft failure if they fulfilled the above criteria and this was added as a covariate in the Cox model.

Missing data regarding area socio‐economic deprivation were ascertained in only (0.6%) of the data and were adjusted for as dummy variables in the models as required (assuming not missing at random). Survival analyses were performed by generation of Kaplan–Meier curve estimates. A *P *< 0.05 was considered statistically significant in the analysis. Data were analyzed using Stata SE 14 (Stata Statistical Software: College Station, TX: Stata Corp LP).

## Results

Between 2003 and 2013, there were 21,371 patients in England who had a kidney transplant recorded in Hospital Episode Statistics (HES). After excluding 2878 patients, due to missing demographic data and patients who had multi‐organ transplants, we were left with 18,493 patients over the age of 18 for analysis. Over the same time span between 2003 and 2013, there were 12,373 patients in NYS who had a kidney transplant recorded in the SPARCS. After excluding 771 patients, due to missing demographic data and recipients of multi‐organ transplants, we were left with 11,602 patients over the age of 18 for analysis. Median follow up after kidney transplantation in England and New York State was 6.3 years and 5.5 years respectively. Total patient‐years of follow up for kidney allograft recipients in England and NYS was 90,655 and 67,743, respectively.

### Baseline demographics

A comparison of baseline demographics between English and NYS kidney allograft recipients is given in Table 1. Significant differences were noted in baseline variables between the two cohorts, with the NYS cohort more likely to be older and non‐White. Of interest, English kidney allograft recipients were more likely to have had pretransplant cancer than NYS patients (5.6% vs. 3.5% respectively, *P *< 0.001).

**Table 1:** Comparison of baseline demographics between England and New York State

		England (%)	NYS (%)	*P*‐value
Total		18493	11602	
Age	Mean	47.61	50.88	<0.001
Post‐transplant hospital stay	Median	8 (6–13)	5 (4–7)	<0.001
Sex	Male	11370 (61.48%)	7033 (60.62%)	
	Female	7123 (38.52%)	4569 (39.38%)	0.136
Type of Donor	Living	6706 (36.26%)	3977 (34.28%)	
	Deceased	11459 (61.96%)	5326 (45.91%)	
	Unknown	328 (1.77%)	2299 (19.82%)	<0.001
Operation Year	2003	949 (5.13%)	835 (7.20%)	
	2004	1362 (7.36%)	957 (8.25%)	
	2005	1346 (7.28%)	1119 (9.64%)	
	2006	1471 (7.95%)	1226 (10.57%)	
	2007	1532 (8.28%)	1212 (10.45%)	
	2008	1763 (9.53%)	1174 (10.12%)	
	2009	1893 (10.24%)	1113 (9.59%)	
	2010	1938 (10.48%)	1034 (8.91%)	
	2011	1908 (10.32%)	1070 (9.22%)	
	2012	2038 (11.02%)	1041 (8.97%)	
	2013	2293 (12.40%)	821 (7.08%)	<0.001
Pretransplant Cancer		1033 (5.59%)	408 (3.52%)	<0.001
Ethnic Group	White	14508 (78.45%)	6202 (53.46%)	
	Black	1215 (6.57%)	2660 (22.93%)	
	Other	2770 (14.98%)	2740 (23.62%)	<0.001

### Comparison of cancer incidence between England and NYS

English kidney allograft recipients were more likely to be admitted to hospital post‐transplant with a new cancer diagnosis compared to NYS kidney allograft recipients. In total, 10.6% (*n *= 1965) of English recipients and 7.3% (*n *= 846) of NYS recipients were admitted with new development of cancer (*P *< 0.001). There was no difference in the median time to cancer diagnosis between England (3.7 years, IQR: 1.7–6.2) and NYS (3.5 years, IQR: 1.6–6.1) respectively (*P *= 0.288). Table 2 highlights the differences in cancer‐specific incidence post kidney transplantation comparing England to NYS (Table S1. identifies cancer incidence within first year after kidney transplantation). The most notable difference between cohorts worthy of highlighting was significantly higher incidence of melanoma skin cancer in England versus NYS, which may reflect the lower frequency of non‐Whites in the English kidney transplant cohort.

**Table 2:** Incidence of cancer comparing England versus New York State after kidney transplantation

Post‐transplant type of cancer	England (%)	NYS (%)
Lip, oral cavity and pharynx	33 (0.18)	16 (0.14)
Digestive organs	119 (0.64)	113 (0.97)
Respiratory and intrathoracic organs	73 (0.39)	108 (0.93)
Bone and articular cartilage	a	9 (0.08)
Melanoma and other malignant neoplasms of skin	848 (4.59)	53 (0.46)
Mesothelial and soft tissue	29 (0.16)	6 (0.05)
Breast	85 (0.46)	30 (0.26)
Female Genital Organs	37 (0.20)	21 (0.18)
Male Genital Organs	83 (0.45)	84 (0.72)
Kidney	113 (0.61)	97 (0.84)
Ureter	a	a
Bladder	51 (0.28)	28 (0.24)
Other and unspecified urinary organs	a	a
Eye, brain, and other parts of the central nervous system	11 (0.06)	9 (0.08)
Thyroid and other endocrine gland	16 (0.09)	19 (0.16)
Ill‐defined, secondary, and unspecified sites	192 (1.04)	86 (0.74)
Lymphoid, hematopoietic, and related tissue	262 (1.42)	156 (1.34)
Independent multiple sites	a	a

a Numerically too few for data to be provided.

### Comparison of all‐cause mortality and kidney transplant rejection/failure between England and NYS

Kidney allograft recipients in England overall had lower all‐cause mortality compared to their NYS counterparts at all time points investigated in this analysis; during the immediate postoperative stay (0.7% vs. 1.0% respectively, *P *= 0.011), after 30‐days (0.9% vs. 1.8% respectively, *P *< 0.001) and after 1‐year post kidney transplantation (3.0% vs. 5.1% respectively, *P *< 0.001). However, for kidney allograft recipients specifically who developed post‐transplant cancer, there was no difference in 1‐year mortality between England versus NYS (2.6% vs. 3.4% respectively, *P *= 0.223). Too few in‐hospital or 30‐day deaths occurred for data to be released and analyzed for both English and NYS cohorts.

**Figure 1:**
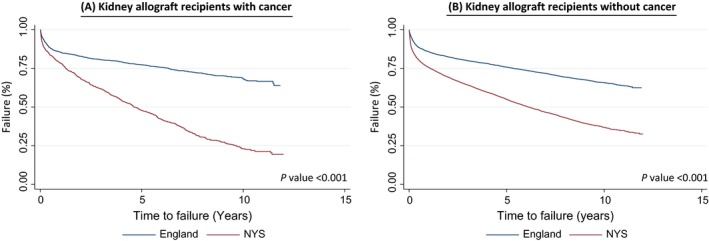
Kaplan–Meier plot of time to kidney transplant rejection/failure for kidney allograft recipients with or without post‐transplant cancer in England versus New York State.

Kidney allograft recipients in England had lower risk for kidney transplant rejection/failure compared to their NYS counterparts. For kidney allograft recipients with post‐transplant cancer, kidney transplant rejection/failure was less common in England versus NYS (26.9% vs. 69.0% respectively, *P *< 0.001) (see Fig. 1A). For kidney allograft recipients without post‐transplant cancer, the risk for kidney transplant rejection/failure remained lower in England versus NYS (25.4% vs. 50.8% respectively) is shown in the unadjusted Kaplan–Meier plots (see Fig. 1B).

### Adjusted analysis

Table 3 highlights the multivariable logistic regression analysis looking at 1‐year mortality after kidney transplantation in both English and NYS cohorts after adjustment for important covariables. It demonstrates patients admitted with cancer within the first year post‐transplantation was a strong predictor for 1‐year all‐cause mortality in both England (Odds Ratio 5.0 [95% CI: 3.6–6.9], *P *< 0.001) and NYS (Odds Ratio 3.3 [95% CI: 2.0–5.6], *P *< 0.001). After incorporating all patients into a combined cohort, even after adjustment for cancer incidence within the first year, kidney allograft recipients from NYS had increased odds for 1‐year all‐cause mortality compared to English counterparts (Odds Ratio 1.47 [95% CI: 1.29–1.67], *P *< 0.001).

**Table 3:** Logistic regression analysis of 12‐month mortality in England versus New York State with cancer diagnosis within first year

Variable		Odds Ratio (95% CI)	*P*‐value
England			
Age	<50	1 (baseline group)	
	50+	3.76 (3.07,4.61)	<0.001
Sex	Male	1 (baseline group)	
	Female	1.06 (0.89,1.27)	0.495
Ethnicity	Black	1 (baseline group)	
	Other	1.27 (0.85,1.90)	0.234
	White	0.94 (0.65,1.34)	0.728
Number of readmissions	0	1 (baseline group)	
	1 to 3	0.70 (0.56,0.86)	0.001
	3 +	0.42 (0.34,0.52)	<0.001
Cancer within 1 year after	Yes	5.01 (3.64,6.90)	<0.001
	No	1 (baseline group)	
New York State			
Age	<50	1 (baseline group)	
	50+	1.44 (1.21,1.72)	<0.001
Sex	Male	1 (baseline group)	
	Female	1.05 (0.89,1.25)	0.762
Ethnicity	Black	1 (baseline group)	
	Other	0.74 (0.59,0.94)	0.064
	White	0.67 (0.55,0.82)	<0.001
Number of readmissions	0	1 (baseline group)	
	1 to 3	0.89 (0.73,1.09)	0.261
	3 +	0.46 (0.37,0.58)	<0.001
Cancer within 1 year after	Yes	3.33 (1.99,5.57)	<0.001
	No	1 (baseline group)	

We performed Cox regression analysis on kidney transplant rejection/failure due to data availability regarding time to diagnosis. Regardless of whether the analysis focused on kidney allograft recipients with or without post‐transplant cancer (Tables 4 and 5 respectively), kidney allograft recipients in NYS demonstrated higher hazard ratios for development of kidney transplant rejection/failure compared to England.

**Table 4:** Cox regression analysis of kidney transplant rejection/failure for noncancer patients

		Hazard Ratio (95% CI)	*P*‐value
Age	<50	1 (baseline group)	
	50 +	0.87 (0.84,0.91)	<0.001
Sex	Male	1 (baseline group)	
	Female	1.04 (0.99,1.08)	0.096
Ethnic Group	White	1 (baseline group)	
	Black	0.75 (0.70,0.80)	<0.001
	Other	0.84 (0.79,0.89)	<0.001
Type of Donor	Alive	1 (baseline group)	
	Dead	1.33 (1.27,1.39)	<0.001
	Unknown	0.99 (0.92,1.07)	0.86
Diabetes		1.12 (1.06,1.17)	<0.001
Acute MI		1.05 (0.98,1.13)	0.19
CVF		1.11 (1.02,1.20)	0.016
PVD		1.16 (1.07,1.25)	<0.001
CHF		1.12 (1.05,1.19)	<0.001
Year	Pre 2007	1 (baseline group)	
	Post 2007	1.20 (1.14,1.26)	<0.001
Country	England	1 (baseline group)	
	NYS	2.20 (2.10,2.30)	<0.001

**Table 5:** Cox regression analysis of kidney transplant rejection/failure for cancer patients

		Hazard Ratio (95% CI)	*P*‐value
Age	<50	1 (baseline group)	
	50 +	0.87 (0.76,1.00)	0.044
Sex	Male	1 (baseline group)	
	Female	0.93 (0.82,1.06)	0.288
Ethnic Group	White	1 (baseline group)	
	Black	0.92 (0.73,1.17)	0.506
	Other	0.92 (0.76,1.11)	0.365
Type of Donor	Alive	1 (baseline group)	
	Dead	1.08 (0.94,1.24)	0.287
	Unknown	0.85 (0.69,1.04)	0.119
Diabetes		1.21 (1.06,1.39)	0.007
Acute MI		0.93 (0.76,1.15)	0.523
CVF		1.11 (0.87,1.41)	0.403
PVD		1.08 (0.86,1.35)	0.497
CHF		1.06 (0.88,1.27)	0.561
Year	Pre 2007	1 (baseline group)	
	Post 2007	1.52 (1.33,1.74)	<0.001
Country	England	1 (baseline group)	
	NYS	3.16 (2.74,3.65)	<0.001

Sensitivity analysis was conducted after excluding cancers that had occurred within the first 2 years’ post‐transplant to exclude the possibility of reverse causality. Cancer incidence rate (adjusted for age and sex) in England was 90.6 versus 64.7 cases per 1000 patients for the whole cohort and after excluding cancers within the first 2 years, respectively. Cancer incidence rate (adjusted for age and sex) in NYS was 58.7 versus 39.9 cases per 1000 patients for the whole cohort and after excluding cancers within the first 2 years, respectively. Therefore, there was little difference in comparative incidence ratio for England versus NYS when comparing the whole cohort or early cancers excluded (1.54 [95% CI: 1.48–1.60] and 1.62 [95% CI: 1.55–1.69], respectively.

## Discussion

To the best of our knowledge, this study is the first comparative analysis of cancer‐related epidemiology after kidney transplantation between different countries. This population‐cohort analysis, comparing kidney allograft recipients between England and NYS, suggests caution in extrapolating results between different patient cohorts but also raises some important topics for further discussion.

It is well recognized that kidney transplantation is associated with an increased incidence of cancer compared to other stages of the renal disease spectrum 8, with comparable burden to other immune deficiency states such as HIV/AIDS 21. Population‐cohort analyses from different countries suggest similar post kidney transplantation risk for cancer regardless of geography. For example, Collett and colleagues explored cancer incidence after solid‐organ transplantation in the United Kingdom by linking data between NHS Blood and Transplant to various cancer registries between 1980 and 2007 11. They demonstrated a twofold increased risk for incidence of post‐transplant cancer compared to the general population and particularly high risk for nonmelanoma skin cancer 11. This finding was surprisingly similar to the twofold increase in risk for cancer incidence identified in the United States population by Kasiske and colleagues, who linked patients from the United States Renal Data System (USRDS) to Medicare billing claims to detect the occurrence of cancer 7.

However, disparate results have been published regarding the risk for cancer‐related mortality between England and the United States. For the latter, Kiberd et al. identified 1937 malignancy‐related death amongst 164,078 first kidney allograft recipients recorded in the USRDS between January 1990 and December 2004 3. They demonstrated no difference between the expected and observed cancer mortality rate, with equivalent standardized cancer mortality ratios with the general population, which the authors speculate could be attributed to the numerous competing risks for death that exist post‐transplantation 3. This contrasts with results from an English population‐cohort study by Farrugia and colleagues, who identified cancer‐related mortality (as recorded by death certification) to be elevated for kidney allograft recipients in England (2001–2012 cohort) compared to the general population after competing risk analysis 4. It is important to identify numerous epidemiological differences between these population‐cohort studies including disparate time periods, lack of comparable baseline demographics and different transplant practice. However, subsequent publications from Canada support the findings from Farrugia and colleagues linking increased cancer‐related mortality for kidney allograft recipients 5. Our study lacked cancer‐specific mortality data, which is a limitation, but all‐cause mortality rates for kidney allograft recipients was lower in England versus NYS. This is consistent with data demonstrating superior long‐term outcomes after kidney transplantation in Europe compared to the United States; for example, Ojo and colleagues found kidney allograft recipients in the United States had more than double the long‐term hazard for death with graft function (regardless of whether cause of end‐stage kidney disease was diabetes or not) 22.

While all‐cause mortality was significantly lower for English versus NYS kidney allograft recipients in our analysis, we did not identify any increased risk for death post‐transplantation among those who developed cancer. Epidemiological studies of the general population have consistently given the interpretation of superior cancer survival in the United States compared to countries like the United Kingdom, based upon studies looking at global cancer incidence and mortality 8. For example, the worldwide population‐based study entitled CONCORD highlighted superior 5‐year survival rates for prostate cancer patients diagnosed in the United States (91.9%) compared to the United Kingdom (51.1%) 23. However, such crude interpretations of the raw data belie a more complicated scenario. For example, the use of prostate hormone antigen (PSA) has been more prevalent in the United States compared to places like Europe. This results in better detection of prostate cancer in the United States, and subsequently one of the highest recorded rates of prostate cancer, but inevitably labels some men with prostate cancer who will never die from the disease. Recent studies have cast doubt on the ubiquitous use of PSA 24, 25 and published recommendations against the routine use of PSA 26 have led to subsequent falls of PSA testing in the United States 27. The efficacy of cancer screening from a transplantation perspective remains even more unproven than the general population 28. More recent data from the CONCORD‐2 study continues to show wide differences in cancer‐linked survival globally that are attributed to differences in access to early diagnosis and optimum treatment 29. Considering kidney allograft recipients remain under close monitoring under the care of transplant nephrologists, we can speculate that such lifelong surveillance confounds any major difference for a transplant cohort. Our observation of no difference in all‐cause mortality for kidney allograft recipients with post‐transplant cancer (in contrast to the overall kidney allograft recipient cohort) hints at differential care for this select cohort of patients in NYS but this requires further analysis of patient‐level data at a more granular basis before drawing any firm conclusions. However, attempting to explain difference in outcomes for our cohorts, which is likely secondary to multi‐factorial reasons, is beyond the scope of this publication and would be too crude with the current dataset for definitive investigation. Differences in health care funding, services and delivery likely all confound a formal comparison of cohorts between England and NYS. For example, studies comparing healthcare systems in the United Kingdom with elsewhere, including the United States, have shown differences in expenditure (lower in England) 30 and cost effectiveness (lower in United States) 31. However, it should be noted that cancer‐related mortality has been reducing over the last 10–15 years in both England and the United States 32, 33 and our differences in outcome must be interpreted against this background.

English kidney allograft recipients admitted for transplantation were more likely to have a pretransplant diagnosis for cancer in our analysis, which may be linked to the increased incidence of post‐transplant cancer seen in England post‐transplantation (although we lacked data to show if this was recurrent or new onset cancer). Facilitating kidney transplantation for more patients with previous cancer may be the most appropriate clinical course of action. For example, Viecelli and colleagues analyzed the Australian and New Zealand Dialysis and Transplant Registry, which highlighted recurrent and second primary cancers were infrequent after kidney transplantation among patients with a previous history of cancer 34. In addition, a history of pretransplant cancer did not have an additive effect on the cancer‐specific and overall survival of kidney transplant recipients who developed post‐transplant cancer 34. However, this contrasts sharply with findings from Farrugia and colleagues who demonstrated pretransplant cancer in an English kidney transplant cohort had the strongest hazard for post‐transplant cancer‐related mortality (Hazard Ratio 7.653, 95% confidence interval 4.231–13.844, *P *< 0.001) 4. This discordance again highlights the difficulty in translating data between different kidney transplant cohorts and the need for more global epidemiological comparisons of post‐transplant complications (like cancer) to improve our understanding.

This study has several limitations which must be acknowledged for both accurate interpretations of our data and to ensure targeted opportunities for further clinical research. Epidemiological studies such as these are reliant upon extraction of data from regional or national registries, which come with the usual caveats attributable to the accuracy and completeness of registry data. We are also being presumptive that NYS data is reflective of the wider population of the United States, which may not be accurate from both a demographic and transplant practice perspective, and our data should be interpreted specifically as a comparison of data between England and NYS. While data linkage allowed us to extract all‐cause mortality for all our patients, we were unable to obtain cause of death. This would be important to ascertain the rates of cancer‐related and non‐cancer‐related mortality after a diagnosis of post‐transplant cancer is made and how this differs from the general population. In addition, we relied about coding diagnoses for kidney transplant rejection/failure due to the inability to link our data to the specific UK‐ or US‐based Transplant Registries, which would have made our kidney transplant outcomes more robust. Similar limitations exist for extracting cancer data from administrative registries, which has recently been shown to be inferior to dedicated cancer registries in the United States 35. There are lots of biases to make a direct comparison difficult to interpret (e.g., ethnicity, recipient age, donor profiles). Finally, we lacked more granular patient‐level data on important variables including smoking status, immunosuppression, pretransplant cancer histology etc. The ability to extract more data, and to link datasets to create more comprehensive databases, will allow the minimization of confounding and hopefully can provide a strong platform from which to conduct meaningful post‐transplant epidemiological analyses to guide clinical management and patient counseling. This is especially important with regard to immunosuppression, which is one of the major modifiable risk factors for the development of cancer after kidney transplantation.

To conclude, our contemporaneous comparison of kidney allograft recipients between England and New York State demonstrates significant differences relating to cancer‐linked epidemiology at both baseline demographics and post‐transplant outcomes between the two cohorts. Our analysis suggests caution in translating data between countries and the need for more global epidemiological studies to ascertain aspects of care that differ between countries. However, this work does not attempt to tackle the underlying reasons for these differences, which is the subject of ongoing work. Further work focusing on these differences in surveillance, diagnosis and management of cancer after transplantation is critical to develop consensus opinion concerning best practice. By doing so, we can aim to optimize the risk attenuation and clinical management of cancer for kidney allograft recipients globally and ultimately work towards prolonging both patient and kidney allograft survival.

## Conflict of Interest

No relevant conflict of interest for any author.

## Supporting information


**Table S1.** Cancer development within first year after kidney transplantation.
