# Supplementary material for: Cancer‐related outcomes in kidney allograft recipients in England versus New York State: a comparative population‐cohort analysis between 2003 and 2013
Source: Cancer Med. 2017 Jan 30;6(3):563–71. doi: 10.1002/cam4.1015 (PMC5345656; doi:10.1002/cam4.1015)
Supplement: Supplementary file 1 — Table S1. Cancer development within first year after kidney transplantation. [file CAM4-6-563-s001.docx]

**Supplementary Table 1. Cancer development within first year after kidney transplantation**

| **Cancer within 1 year** | | |
| --- | --- | --- |
| **Location** | **England** | **NYS** |
| Bone, articular cartilage | 0 | * |
| Eye, brain, CNS | * | 0 |
| Breast | 13 (0.07%) | * |
| Digestive organs | 23 (0.12%) | 14 (0.08%) |
| Female genital organs | 7 (0.04%) | 0 |
| Kidney | 39 (0.21%) | 13 (0.07%) |
| Lip, oral cavity, pharynx | * | * |
| Lymphoid, haematopoietic | 72 (0.39%) | 41 (0.22%) |
| Male genital organs | 21 (0.11%) | 25 (0.14%) |
| Mesothelial, soft tissue | 6 (0.03%) | 0 |
| Chest and intra-thoracic | 15 (0.08%) | 11 (0.06%) |
| Ill-defined, secondary and unspecified sites | 40 (0.22%) | 7 (0.04%) |
| Melanoma and other malignant neoplasms of skin | 102 (0.55%) | 6 (0.03%) |
| Thyroid and other endocrine gland | * | * |
| Bladder, Ureter | 21 (0.11%) | * |

*Numerically too small to identify
